# WWOX and Its Binding Proteins in Neurodegeneration

**DOI:** 10.3390/cells10071781

**Published:** 2021-07-14

**Authors:** Che-Yu Hsu, Kuan-Ting Lee, Tzu-Yu Sun, Chun-I. Sze, Shenq-Shyang Huang, Li-Jin Hsu, Nan-Shan Chang

**Affiliations:** 1 Laboratory of Molecular Immunology, Institute of Molecular Medicine, College of Medicine, National Cheng Kung University, Tainan 70101, Taiwan; bryanhsuks@gmail.com (C.-Y.H.); ayta860404@gmail.com (K.-T.L.); elisa128happy@gmail.com (T.-Y.S.); 2 Department of Cell Biology and Anatomy, College of Medicine, National Cheng Kung University, Tainan 70101, Taiwan; chuni.sze@gmail.com; 3 Graduate Program of Biotechnology in Medicine, Institute of Molecular and Cellular Biology, National Tsing Hua University, Hsinchu 300044, Taiwan; louis19862002@gmail.com; 4 Department of Medical Laboratory Science and Biotechnology, College of Medicine, National Cheng Kung University, Tainan 70101, Taiwan; lijin.hsu@gmail.com; 5 Graduate Institute of Biomedical Sciences, College of Medicine, China Medical University, Taichung 40402, Taiwan; 6 Department of Neurochemistry, New York State Institute for Basic Research in Developmental Disabilities, New York, NY 10314, USA

**Keywords:** tumor suppressor, p53, WWOX, TRAPPC6A, TIAF1, SH3GLB2, Alzheimer’s disease, neurodegeneration, functional antagonism

## Abstract

WW domain-containing oxidoreductase (WWOX) is known as one of the risk factors for Alzheimer’s disease (AD), a neurodegenerative disease. WWOX binds Tau via its C-terminal SDR domain and interacts with Tau phosphorylating enzymes ERK, JNK, and GSK-3β, and thereby limits AD progression. Loss of WWOX in newborns leads to severe neural diseases and early death. Gradual loss of WWOX protein in the hippocampus and cortex starting from middle age may slowly induce aggregation of a protein cascade that ultimately causes accumulation of extracellular amyloid beta plaques and intracellular tau tangles, along with reduction in inhibitory GABAergic interneurons, in AD patients over 70 years old. Age-related increases in pS14-WWOX accumulation in the brain promotes neuronal degeneration. Suppression of Ser14 phosphorylation by a small peptide Zfra leads to enhanced protein degradation, reduction in NF-κB-mediated inflammation, and restoration of memory loss in triple transgenic mice for AD. Intriguingly, tumor suppressors p53 and WWOX may counteract each other in vivo, which leads to upregulation of AD-related protein aggregation in the brain and lung. WWOX has numerous binding proteins. We reported that the stronger the binding between WWOX and its partners, the better the suppression of cancer growth and reduction in inflammation. In this regard, the stronger complex formation between WWOX and partners may provide a better blockade of AD progression. In this review, we describe whether and how WWOX and partner proteins control inflammatory response and protein aggregation and thereby limit AD progression.

## 1. Brain Protein Aggregation Starts from Middle Age

Alzheimer’s disease (AD) is the most common cause (60–75%) of dementia and an age-related neurodegenerative disease. Occurrence of AD is frequently seen in patients over 65 years old—the so called late-onset AD. For early-onset AD, the symptoms may start in patients in their 30s or 40s due to gene mutations inherited in an autosomal dominant fashion [1,2,3]. The early-onset is approximately 1–2% of AD. For example, gene mutations of amyloid precursor protein (APP) on chromosome 21, presenilin 1 (PSEN1) on chromosome 14, and presenilin 2 (PSEN2) on chromosome 1 are associated with early-onset AD [1,2,3]. It is generally agreed that protein aggregation plays a key role in the pathogenesis of age-related AD progression. Indeed, protein aggregates are present in the brains of healthy middle-aged individuals, suggesting that AD progression may start gradually in middle-aged humans [4,5,6,7]. Currently, there is insufficient knowledge regarding how AD progression starts from middle age.

Extracellular amyloid beta (Aβ) plaques around the neurons and intracellular neurofibrillary tangles (NFTs) caused by hyperphosphorylated tau proteins are considered the key markers of AD [1,2,3]. Significant reduction in the level of acetylcholine (ACh) neurotransmitter is frequently observed in AD [1,2,3]. Aggregate formation of tau and Aβ levels in the brains are very low in middle-aged healthy humans of 40 to 70 years old [4,5,6,7]. Aβ and tau aggregates are able to invoke neuronal death and block neurogenesis and learning and memory capabilities in old AD patients.

## 2. Spread of Pathogenic Tau and Aβ in the Brain

Pathogenic tau, Aβ, and other proteins can be spread by transmitting from one neuron to another by exocytosis or via synapses or extracellular vesicles (EV) in the central nervous system (CNS), or transferring to the CNS from the peripheral nervous system [8,9,10,11,12,13]. The transmission results in amplification of the pathogenic proteins. Conceivably, spreading and propagation contribute to tau and Aβ aggregation and pathogenesis in AD patients [11]. Once uptaken by neurons, for example, oligomeric tau becomes aggregated and accumulates intracellularly, and the aggregated tau proteins may lead to neuronal death eventually if the proteasome-based protein degradation system is severely damaged [10,11,12,13]. These toxic proteins cause abnormal postsynaptic function and induce neuronal dysfunction leading to cognitive impairments. It is unclear when pathological protein transmission between neurons starts to occur during a human life span.

It appears that hyperphosphorylated tau protein captured in the EV is conformationally altered and is pathogenic. Tau binds and stabilizes microtubules. A small portion of intracellular tau is released to the extracellular space under physiological conditions [14]. Tau is found in the body fluids such as cerebrospinal fluid and blood. Spatiotemporal spreading and distribution of pathogenic tau correlates with cognitive decline in AD patients [8,9,10,11]. It is postulated that when tau is misfolded in the neurons, it can be released from one neuron and transmitted to another. Rapid transmission of pathogenic tau in a prion-like manner among neurons leads to diseased conditions and eventual neuronal death.

Presumably, tau undergoes misfolding during the period from middle to old age, and then the misfolded proteins are transmitted among neurons prior to the appearance of AD symptoms. Conceivably, misfolded tau proteins are the pathological seeds for native tau to merge and become misfolded. Phosphorylation in many specific amino acid residues in tau causes tau aggregation. Acetylated tau promotes memory loss due to disruption of the formation of spatial memories [15]. Targeting acetylation in tau is considered as a therapeutic strategy to restore memory loss.

## 3. Protein Instability and Aggregation and Induced Chronic Brain Inflammation with Age

There is an age-dependent increase in protein instability and aggregation [16,17,18]. The aggregated proteins can ultimately lead to the formation of Aβ plaques [17]. When protein aggregation occurs in middle-aged *C. elegans*, no significant aggregation of amyloid β occurs [17]. Similarly, tau and Aβ aggregates are hard to detect in the brains of middle-aged healthy individuals [4,5,6,7].

The upstream pathway(s) that causes Aβ aggregation is largely unknown. Furthermore, the deciding factors rendering protein aggregation are not well defined. If protein aggregation starts in middle age and goes on very slowly year by year, it would be very difficult to identify and characterize the aggregation initiator(s) and the downstream protein aggregation cascade(s). Aggregated protein complexes suppress proteasomal function [19], and induce chronic inflammations due, in part, to upregulated inflammatory cytokines and activated microglial cells before the late-onset of AD [4,5,6,20]. The chronic inflammation eventually induces accumulation of Aβ plaques and tau tangles to generate the AD symptoms at old age.

## 4. TGF-β Induces Intracellular Protein Aggregation

Transforming growth factor beta (TGF-β) orchestrates the formation of extracellular matrix and controls cell growth or death. TGF-β strongly inhibits the growth of epithelial and endothelial cells [5,21,22]. TGF-β enhances cellular protein aggregation, which affects cellular senescence and stem cell aging [5,21]. TGF-β levels are upregulated in the neocortex of AD and related dementia [23]. In contrast, TGF-β/Smad signaling is significantly downregulated in AD patients [21,22,23,24,25].

## 5. Protein Aggregates of TRAPPC6A, TIAF1, and SH3GLB2 in the Middle-Aged Hippocampi

We have demonstrated the presence of TGF-β-regulated protein aggregation in the hippocampi of middle-aged normal human brains. Three proteins have been identified, which are TGF-β-induced antiapoptotic factor (TIAF1), trafficking protein particle complex subunit 6A (TRAPPC6A or TPC6A), and SH3 domain-containing GRB2 such as endophilin B2 (SH3GLB2) [4,5,6,26,27,28,29,30]. These proteins are responsive to TGF-β1-induced polymerization [4]. The aggregated TIAF1 and TPC6A proteins, for example, are found in the hippocampi and cortices of middle-aged normal humans (40 to 75 years old), and do not undergo degradation with age [5,6].

Transiently overexpressed TIAF1 polymerizes and causes degradation of membrane amyloid precursor protein (APP), followed by generation of Aβ and amyloid fibrils in vitro [4,5,6,26,27,28,29,30]. In parallel, undegradable TIAF1 aggregates progressively cause accumulation of Aβ, fibrils, and plaques in the brain with age [4,5,6]. Polymerized TIAF1 binds Smad2/3/4 to prevent nuclear translocation, and thereby restricts SMAD-mediated gene transcription in vitro [4,5,6,26,27,28,29,30]. The observations suggest that downregulation of TGF-β/Smad signaling in old AD patients is due, in part, to TIAF1 aggregation.

## 6. WWOX in Neural Diseases

In the following sections, we will discuss how tumor suppressor WW domain-containing oxidoreductase (WWOX, FOR, or WOX1) limits protein aggregation [31,32,33,34,35,36,37]. Most recently, WWOX is regarded as one of the five newly-discovered risk factors for AD [38].

### 6.1. WWOX-Interacting Partners for AD

**Figure 1:**
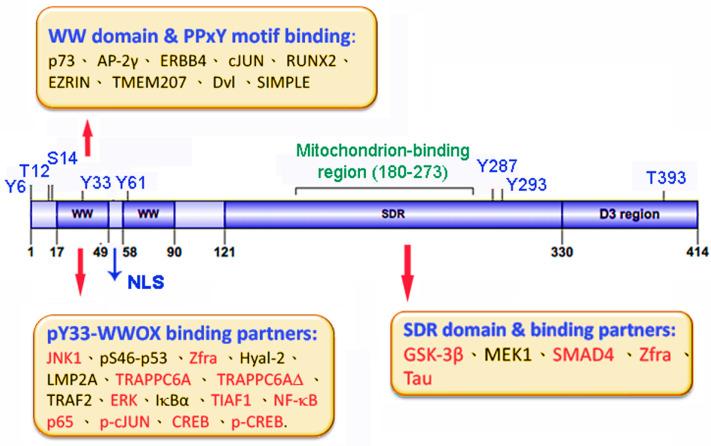
WWOX physically binds proteins via the *N*-terminal first WW domain and the *C*-terminal SDR domain. Upon phosphorylation at Tyr33, pY33-WWOX acquires a significant increase in the numbers of binding partners. Phosphorylation sites at Ser14, Tyr33, and Tyr287 have been documented [52,53,54]. Other phosphorylation sites need further investigation. See legends in the text for details. NLS = nuclear localization signal (KRKR).

WWOX protein possesses two *N*-terminal WW domains containing conserved tryptophan residues, a nuclear localization signal located between the WW domains, a *C*-terminal short-chain alcohol dehydrogenase/reductase domain (SDR), and a proapoptotic *C*-terminal tail termed D3 [35,36,37,38,39,40] (Figure 1). WWOX exhibits numerous functions and participates in many signaling pathways [31,32,33,34,35,36,37]. There are three types of binding interactions between WWOX and its protein partners. First, the *N*-terminal first WW domain binds PPxY or PPPY motif in the target proteins [32,33,39,40] (Figure 1).

Second, when cells are exposed to stress stimuli, WWOX undergoes Tyr33 phosphorylation [32] (Figure 1). pY33-WWOX has an expanded capability in binding protein partners. Binding of pY33-WWOX with the target proteins does not depend upon the PPxY (or PPPY) motif. Notably, many of the pY33-WWOX-interacting proteins (marked in red) participate in neuropathological events in vivo. For example, pY33-WWOX binds JNK and ERK and blocks hyperphosphorylation of tau by these enzymes [41]. pY33-WWOX physically binds TPC6A, TPC6A∆, and TIAF1, and prevents their aggregation in the brain [4,5,6,28,30]. During sciatic nerve dissection, endogenous p-cJUN, p-CREB, and NF-κB p65 bind pY33-WWOX in vivo to undergo nuclear translocation, so as to rescue damaged neurons or induce neuronal death [42].

Zfra (Zinc finger-like protein that regulates apoptosis) is a 31-amino-acid protein that binds WWOX at both the *N*-terminal WW domain and the *C*-terminal SDR domain [43]. Zfra is a potent inhibitor of protein aggregation in AD progression [28]. pY33-WWOX binds a viral protein LMP2A to block proliferation of Epstein–Barr virus [44]. The binding affinity of pY33-WW1 domain with a p73 peptide is reduced, compared to that of non-phosphorylated WW1 with p73 [45]. In contrast, transiently overexpressed full-length WWOX and p73 have an increased binding, in which WWOX is Y33 phosphorylated [46]. The differences in binding strength are unknown. However, both studies did not check the phosphorylation of endogenous WWOX at Y33 and whether pY33-WWOX interacts with a specific domain in endogenous p73. Furthermore, transient overexpression may cause artificial binding effects.

Third, the SDR domain contains a catalytic site to bind NADPH and contributes to the redox activity of WWOX, which is involved in aerobic metabolism [32,33,34,35,36]. An NSYK (Asn-Ser-Tyr-Lys) motif in the SDR domain is responsible for binding sex steroid hormones such as androgen and estrogen [47,48]. SDR domain binds and blocks GSK-3β-mediated tau hyperphosphorylation and thereby promotes neuronal differentiation [41,49]. TGF-β1 and hyaluronan trigger the signaling of membrane Hyal-2/WWOX/Smad4 to induce cell proliferation or death [36,50,51]. The Hyal-2/WWOX/Smad4 signaling contributes to neuronal death during traumatic brain injury [50].

Finally, tyrosine kinase ACK1 induces polyubiquitination of WWOX to undergo proteasomal degradation [52], which is critical for controlling cell growth. Many WW domain-containing proteins bind PPxY motif-protein targets. This binding leads to subsequent ubiquitination and degradation in the target protein [55]. However, WWOX does not appear to possess such a function.

### 6.2. WWOX Loss or Dysfunction Accelerates Cell Migration: Potential Role in Neuronal Heterotopia

A portion of WWOX is located in the cell membrane [47,50,51,56,57]. When normal or functional WWOX-expressing cells (WWOXf) encounter WWOX-dysfunctional or -deficient metastatic cancer cells (WWOXd), the collectively migrating WWOXf cells force the individually migrating WWOXd cells to undergo retrograde migration [47,56]. WWOXd cells, in return, induce apoptosis of WWOXf cells from a remote distance. We determined that cell surface epitope WWOX286-299 (repl) in WWOXf repels the invading WWOXd to move backward [47]. In contrast, when epitope WWOX7-21 (gre) is exposed, WWOXf greets WWOXd to migrate forward for merging. The gre peptide is potent in blocking cancer growth in vivo [57,58]. We identified a membrane signaling complex, which is composed of WWOX, type II TGFβ receptor (TβRII), and Hyal-2 [47]. Specific inhibition of each component by antibody leads to alteration in cell–cell recognition. That is, the membrane complex is crucial in deciding the status of cell–cell recognition, either merging peacefully or killing each other [47].

Neuronal heterotopia or neuronal migration disorder contributes, in part, to the development of seizures [7,59,60], and that WWOX plays a critical in the seizure development [61,62,63,64]. For example, *Wwox* deficiency leads to neurodevelopmental and degenerative neuropathies in newborns [61,62,63,64]. Mechanistically, GSK-3β mediates epileptic seizure activity in mice [61]. Both *Wwox* knockout and heterozygous mice are readily subjected to seizure attack [61]. These mice have WWOXd cells. When cortical neurons possess dysfunctional WWOX protein, these neuronal cells are likely to undergo accelerated migration to the epicortex [61].

### 6.3. WWOX/Hyal-2/Smad4 Signaling in Traumatic Brain Injury

WWOX/Hyal-2 signaling is involved in traumatic brain injury (TBI). During TBI in rats, Hyal-2 and WWOX undergo nuclear translocation, and the Hyal-2/WWOX complex becomes accumulated in the nucleus and causes neuronal death [50]. In vitro analysis revealed that nuclear accumulation of Hyal-2 and WWOX leads to non-apoptotic bubbling cell death (BCD) at 37 °C [36,50,65,66,67]. High-molecular-weight hyaluronan induces BCD when the signaling complex Hyal-2/WWOX/Smad4 is overexpressed [50]. Interestingly, p53 competes with Hyal-2 in binding WWOX. As a result, the p53/WWOX protein complex does not undergo nuclear translocation and no BCD occurs [66]. Instead, the cells undergo blebbing without apoptotic death, suggesting that under stress conditions, p53 may counteract the function of WWOX in blocking cell death.

In principle, WWOXf cells exhibit BCD and functional Ca^2+^ influx in response to UV or apoptotic stress at room temperature [47]. When cells are exposed to chemotherapeutic chemicals, BCD also occurs at 37°C [58]. Hyaluronan-mediated signaling via Hyal-2/WWOX/Smad4 causes BCD at 37°C [36,50]. However, WWOXd cells have a defective or a less efficient system in generating Ca^2+^ influx. WWOXd cells undergo non-apoptotic explosion in response to UV irradiation in room temperature.

### 6.4. WWOX Binds Transcription Factors for Relocating to the Nucleus during Neuronal Damage

In vitro studies showed that pro-survival JNK1 blocks the proapoptotic function of WWOX [53,68]. During the acute phase of sciatic nerve dissection in rats, both JNK1 and WWOX become activated and relocate to the nuclei. Large-sized neurons in the dorsal root ganglion die in 24 hours [42]. Two months later in the chronic phase, concurrent activation of WWOX, CREB, and NF-κB occurs to delay the loss of mall neurons [42]. Apparently, binding of pY33-WWOX with p-CREB or NF-κB is needed to delay the loss of small neurons.

Transiently overexpressed WWOX frequently sequesters transcription factors in the cytoplasm, and thereby blocks their transcription for prosurvival proteins in the nucleus of cancer cells in vitro [33,34]. However, the observations do not occur in vivo. As mentioned above, WWOX co-translocates with transcription factors (e.g., cJun, CREB, and NF-kB) to the nuclei to exert biological or pathological function in vivo [42].

## 7. WWOX in Alzheimer’s Disease

WWOX blocks Tau hyperphosphorylation by directly binding Tau via its *C*-terminal SDR domain [41,69]. By direct binding, the SDR domain of WWOX restricts GSK3β-mediated hyperphosphorylation of Tau [41,69]. In contrast, the first WW domain of WWOX binds JNK and ERK, and thereby prevents Tau hyperphosphorylation [41,69]. Together with WWOX, Tau protein supports polymerization of tubulin monomers for microtubule assembly, so as to promote neurite outgrowth [48].

### 7.1. WWOX in Neural Development and Neural Diseases

During mouse embryonic development, WWOX protein is highly expressed in the neural crest-derived structures such as cranial and spinal ganglia, skin pigment cells, and mesenchyme in the head, indicating the potential involvement of WWOX in neuronal differentiation and maturation [69,70,71,72,73,74,75,76,77]. Genetic deficiency of *WWOX/Wwox* gene causes severe neural diseases (e.g., epilepsy, microcephaly, retinal degeneration, and ataxia), metabolic disorders (including lipid, cholesterol, and glucose metabolism), and early death in newborns [60,61,62,63,69,70,71,72,73,74,75]. Chronic inflammation in the brain occurs due to increased activation of GSK-3β for causing epileptic seizure, and upregulation of microglia cells and astrocytes and reduced GABG-ergic inhibitory interneurons in the brain cortex and hippocampus [61]. Together, WWOX deficiency in newborns suffer the disorder of sex differentiation (DSD), spinocerebellar ataxia (SCA), early infantile epileptic encephalopathy (EIEE), and *WWOX*-related epileptic encephalopathy (WOREE syndrome) [60,61,62,63,69,70,71,72,73]. WWOX controls neuronal differentiation, and that loss of WWOX induces activation of GSK-3β that contributes to neurodegeneration [49]. Extensive analysis of available databases also revealed that WWOX participates in autism spectrum disorder [71]. However, there are still no effective remedies to cure WWOX-related neural diseases and early death in newborn infants.

### 7.2. WWOX Downregulation and Induction of Protein Aggregation Cascade

We reported that when WWOX protein is downregulated in the hippocampi of middle-aged individuals, brain protein aggregation may start to occur [4,5,6,7]. The aggregation process is slow and may take 20 to 30 years. Ultimately, extracellular amyloid β plaques and intracellular tau tangles are built up in the brain of AD patients approximately at 70-years old or older. Meanwhile, reduction of inhibitory GABAergic interneuron occurs [62].

In *Wwox* knockout mouse, it only takes less than 15 days after birth to let the brain proteins polymerize and aggregate in a cascade-like manner [4,5,6,7,26,28,75]. The sequential protein aggregation starts from TPC6AΔ, then TIAF1 and SH3GLB2, and ultimately amyloid-beta (Aβ) and tau. In *Wwox* heterozygous mice, they exhibit a faster kinetics of AD progression, compared to triple-transgenic mice [28].

Under physiological conditions, TPC6A or TPC6AΔ binds the C-terminal tail of WWOX in the cytoplasm and provides a piggyback ride for WWOX to translocate to the nucleus [4]. The WWOX/TPC6A complex is then dissociated, and TPC6A translocates to the nucleolus, followed by relocating to the mitochondria [4]. The mitochondrion-nucleolus trafficking suggests that TPC6A carries essential materials from the nucleoli to the mitochondria to support the physiological functions of mitochondria. TGF-β1 causes the dissociation of the WWOX/TPC6A complex [4]. Thus, excessive TGF-β1 induces aberrant signaling and trafficking and eventual aggregation of TPC6A or TPC6AΔ. TPC6A is a member of the TRAPP family. The family proteins have been shown to be associated with neural diseases such as AD [78,79].

WWOX prevents the aggregation of TPC6A or TPC6AΔ in the cytoplasm. Without the presence of WWOX, the fluid-phase TPC6AΔ acquires S35-phosphorylation, binds mitochondria, and undergoes polymerization. TPC6AΔ binds membrane-bound pS37-TIAF1 on the mitochondrial surface. The TPC6AΔ/TIAF1 complex stimulates activation of caspase 3, which leads to Thr688-dephosphorylation of the membrane amyloid precursor protein (APP). APP is then degraded to generate an intracellular domain (AICD) and Aβ. Aβ is secreted into the extracellular matrix to form amyloid fibrils and plaques [4,6,75].

### 7.3. pS14-WWOX Promotes AD Progression

When mice are subjected to traumatic brain injury or sciatic nerve dissection, endogenous WWOX is significantly upregulated and phosphorylated at Tyr33 [36,42,50,80]. pY33-WWOX induces apoptosis from the nucleus, so as to remove damaged cells both in vivo and in vitro.

When pY33-WWOX is downregulated, pS14-WWOX is significantly increased in the lesions of AD hippocampus and cortex [7,28]. pS14-WWOX promotes AD progression in vivo. Suppression of Ser14 phosphorylation by Zfra leads to enhanced degradation of aggregated proteins, reduction in NF-κB-mediated inflammation, and restoration of memory loss in triple transgenic mice for AD [7,28]. Overall, pY33-WWOX prevents neurodegeneration. In stark contrast, pS14-WWOX enhances the progression of neurodegeneration. The underlying signaling pathways that lead to divergent outcomes remain to be established. By the same token, during cancer growth, the proapoptotic pY33-WWOX is downregulated, and pS14-WWOX upregulated [28,81]. Conceivably, transition between pY33 and pS14 phosphorylation in WWOX contributes to the regulation of the progression of AD, cancer and other diseases.

### 7.4. Binding of Endogenous WWOX with Intracellular Partner Proteins In Vivo

The aforementioned pY33-pS14 transition raises the question regarding whether binding of WWOX with its protein partners contributes to the control of disease progression. We determined that Zfra4-10 or WWOX7-21 peptide strongly suppresses the growth of cancer xenografts in mice [57,58]. The growth suppression is associated with intracellular protein complex formation. For example, when mice receive Zfra4-10 or WWOX7-21 peptide, these mice have increased binding of endogenous WWOX with p53 in the spleen, lung, liver, and other organs [57]. This correlates with cancer suppression. Additionally, Zfra4-10 or WWOX7-21 peptide enhances the binding of endogenous WWOX with C1qBP, CD133, p21, JNK1, COX2, p-ERK, Foxp3, and p53 in the spleen. There is also an increased binding of WWOX with Iba1, Oct4, ERK1/2, NF-κB p65, GFAP, and p53 in the lung. Whether the increased binding leads to inhibition of AD progression remains to be established.

In contrast, when Zfra4-10 and WWOX7-21 peptides are in combination in treating mice, binding of endogenous WWOX with protein partners is significantly reduced in many organs, and the cancer xenograft growth is increased [57].

Finally, Zfra4-10 covalently interacts with proteins for rapid degradation in a ubiquitination/proteasome-independent manner [28,81]. Zfra binds the *N*-terminal WW domain and *C*-terminal SDR domain of WWOX [43]. Conceivably, the WWOX/Zfra complex is subjected to degradation and thereby affects the efficacy of Zfra in mitigating AD progression [28].

## 8. p53 and WWOX in AD

Tumor suppressor p53 participates in the AD progression and neurodegeneration [82,83,84]. However, p53 has not been considered as a risk factor for AD [38]. Both p53 and WWOX are essential in maintaining chromosomal integrity and stability [7,33]. WWOX and p53 possess many phosphorylation sites. Phosphorylation in a specific amino acid represents their action to carry out proper duties. p53 tends to undergo mutation and conformational changes. Accordingly, p53 tends to be readily undergoing aggregation, causes chronic inflammation in the brain, and facilitates AD progression [84]. Dysfunctional p53 affects the neuronal physiology, including inflammation, redox homeostasis, normal synaptic function, and amyloid β formation [80,81,82]. During AD progression, chronic inflammatory neurons induce p53 activation, so as to fix damaged DNA. WWOX may play the similar role. However, the inflamed neurons possess tau oligomers due to breakdown of the microtubule network, and this blocks p53 to undergo nuclear translocation. Cytoplasmic accumulation of p53 without degradation leads to aggregation. In the absence of p53 in the nucleus, neurons will undergo cell cycle arrest, DNA damage repair, and apoptosis [84].

### 8.1. Expression of p53 and WWOX and Their Binding Partners in the Human Brains

**Figure 2:**
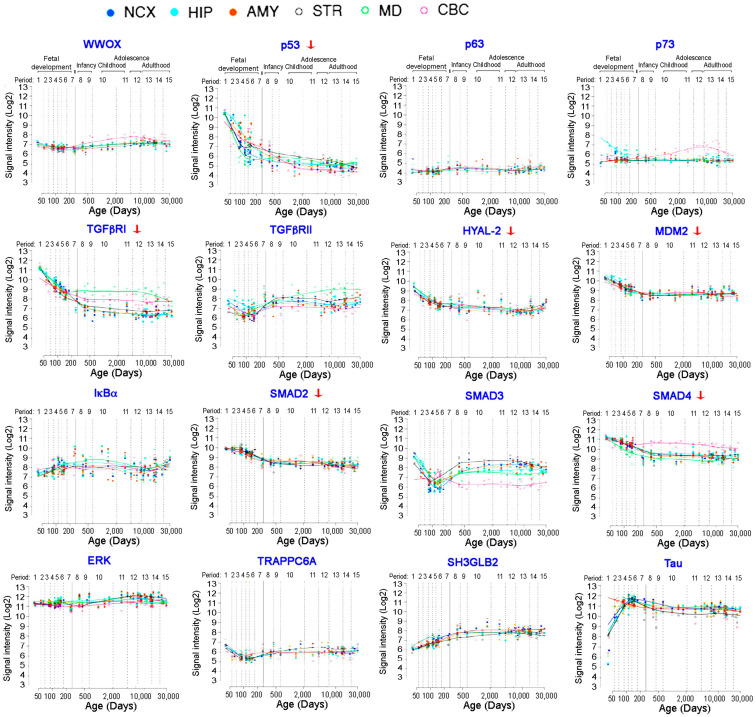
Selected gene expression profiles in the brain with age. Six gene-encoded proteins, including p53, TGFβRI, Hyal-2, MDM2, Smad2 and Smad4, are known to participate in growth suppression and apoptosis. Notably, their gene expression tend to downregulate with age. The gene expression data are from the database of the Human Brain Transcriptome (https://hbatlas.org/pages/hbtd) [85,86]. Gene expression is shown from the brain areas, including cerebellar cortex (CBC), mediodorsal nucleus of the thalamus (MD), striatum (STR), amygdala (AMY), hippocampus (HIP), and 11 areas of neocortex (NCX). Certain genes are not downregulated with age, and their encoded proteins participate in the IκBα/WWOX/ERK [54] and WWOX/TGFβRII signal pathways [47]. However, proapoptotic proteins, including p53, Hyal-2, Smad4, and WWOX, tend to undergo downregulation with age.

We selected 16 WWOX-related gene expression data from the Human Brain Transcriptome (https://hbatlas.org/pages/hbtd; last visit on 25 June 2021) [85,86]. Brain areas examined for gene expression are cerebellar cortex (CBC), mediodorsal nucleus of the thalamus (MD), striatum (STR), amygdala (AMY), hippocampus (HIP), and 11 areas of neocortex (NCX). Notably, the expression of p53 and MDM2 genes are downregulated after birth and the reduction continues in teenage (Figure 2; red arrow). The reduction in gene expression also occurs in MDM2, Hyal-2, Smad3, Smad4, and TGFβRI (Figure 2; red arrows). The levels of other listed gene expression are not downregulated and relatively stable. In the IκBα/WWOX/ERK survival signaling [54], no reduction of gene expression for IκBα, WWOX, and ERK is shown from prenatal to the teenage period. No reduction for WWOX and TGFβRII genes for signaling cell–cell recognition is shown [47]. No downregulation of TRAPPC6A, SH3GLB2 and tau is shown up to the teenage period.

WWOX signals with p53, Smad4, and HYAL-2 for growth suppression, apoptosis, and BCD [26,36,55,76]. The gene expression levels for p53, Smad4, and Hyal-2 are reduced in the brain up to the teenage period. Gene expression for TGFβRI and Smad2 are also downregulated with age, and their encoded proteins can undergo aggregation during AD progression. Presumably, specific genes and encoded protein expression, which participate in the cell growth suppression and apoptosis, tend to undergo downregulation with age.

### 8.2. p53 and WWOX Physical Binding and Induction of Apoptosis

Both p53 and WWOX are guardians of the genome [7,33,87,88]. Under stress conditions, increased binding of p53 with WWOX occurs, in which activated pY33-WWOX complexes with pS15- or pS46-p53 both in vitro and in vivo [31,32,35,36,37,53,80]. Both proteins act synergistically in inducing apoptosis in vitro. Presumably, the stronger the binding of p53 with WWOX, the stronger the complex-mediated cancer suppression and probably inhibition of AD progression. It is not clear whether WWOX binds monomeric or tetrameric p53, or both forms. Whether pS14-WWOX physically binds p53 is unknown. The activated p53/WWOX complex relocates to mitochondria or nuclei to induce apoptosis [31,32,52,80]. Loss of WWOX in cells results in reduced p53 stability and proapoptotic function [80]. Whether MDM2 limits the activity of pS15- or pS46-p53 by complexing with pY33-WWOX is unknown. Transiently overexpressed p53 tends to undergo aggregation and this leads to failure in binding with WWOX.

### 8.3. Estrogen in the p53/WWOX Signaling

When cells are exposed to micromolar amounts of 17-beta estradiol (E2), cell death occurs in a p53/WWOX-dependent manner [89]. Mechanistically, E2 binds the SDR domain of WWOX and then induces activation of both pY33-WWOX and pS15-p53 to cause accumulation of the E2/pY33-WWOX/pS15-p53 complex in the nucleus. The complex leads to apoptosis. Whether this event occurs in vivo has yet to be investigated.

### 8.4. TIAF1, p53, and WWOX Triad in Apoptosis

TIAF1 complexes with p53 and WWOX to form a triad [76]. The TIAF1/p53/WWOX triad is potent in blocking cancer cell migration, anchorage-independent growth, and SMAD promoter activation, and causing apoptosis [76]. However, the role of the TIAF1/p53/WWOX complex in AD is largely unknown. TIAF1 physically binds the first *N*-terminal WW domain of WWOX. Activated p53 binds TIAF1, and WWOX strengthens the p53/TIAF1 complex so as to stabilize the triad. Without p53 activation, no binding interaction occurs between TIAF1 and p53 [76]. Silencing of TIAF1 by siRNA abolishes the nuclear translocation of pS15-p53 in response to UV or etoposide [90]. When TIAF1 is replaced by Smad4 in the p53/WWOX complex, the resulting Smad4/p53/WWOX complex induces membrane blebbing without causing apoptosis [67], suggesting that TIAF1 and Smad4 competitively bind p53 or WWOX, which may affect the extent of cell survival or death.

### 8.5. Extracellular Matrix-Mediated TIAF1 Aggregation and Regulation of SMAD Promoter

When extracellular matrix is altered in the microenvironment, intracellular TIAF1 is upregulated and undergoes aggregation. For example, when any cells are seeded on extracellular matrices derived from other cells, these cells start to increase the intracellular levels of aggregated TIAF1 and Aβ [26,27,91]. The TIAF1/Aβ aggregates further induce the expression of WWOX and Smad4, which in turn builds up TIAF1/Smad4 complex for Aβ accumulation [26,27,91]. For cancer cells, they are able to handle intracellular accumulation of proteins effectively [5,26]. However, the event is detrimental to neurons. For example, cancer cell-derived TIAF1 is toxic to neurons [5,26].

Polymerized TIAF1 binds Smad4 and blocks SMAD promoter activation [26]. When p53 protein is downregulated or absent, TIAF1 undergoes self-polymerization and activates the SMAD-regulated promoter [26]. Interestingly, transiently overexpressed TIAF1 induces WWOX expression, and vice versa [26]. The observations suggest a regulatory control of WWOX for TIAF1 aggregation.

## 9. p53 and WWOX Functional Antagonism In Vivo

**Figure 3:**
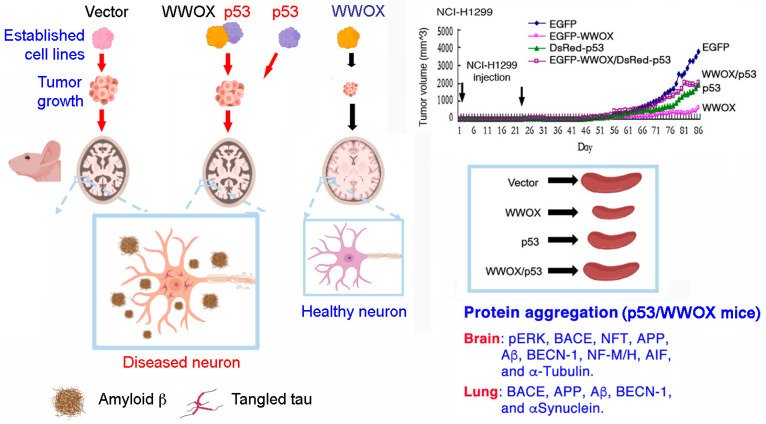
p53 may functionally antagonize the biological functions of WWOX in vivo. Four stable transfectants of NCI-H1299 cells with vector only, WWOX, p53, or WWOX/p53 mammalian expression vectors, were established [76]. Subcutaneous inoculation of these established cells in nude mice revealed that WWOX is most effective in cancer suppression and inhibition of inflammation (splenomegaly), as compared to p53 [76]. p53 inhibits the effect of WWOX in blocking cancer growth. Protein aggregates, including pERK, BACE, APP, Aβ, NFT, and α-tubulin, are found in the brains of p53/WWOX-NCI-H1299 mice, but not in other mice. Protein aggregates in the lung are also observed.

While p53 and WWOX act synergistically in vitro in inducing apoptosis, it is intriguing to find that p53 may functionally antagonize with WWOX in vivo [76]. This functional antagonism leads to increased formation of protein aggregates in the brain and lung, enhanced cancer cell growth, and inflammatory splenomegaly (Figure 3). In our recent study, when non-small cell lung cancer NCI-H1299 cells were stably transfected with p53 or WWOX cDNA, or both cDNA expression constructs, followed by growing in nude mice. Ectopic WWOX alone is most effective in suppressing cancer cell growth than p53 in mice [76]. Moreover, WWOX inhibits cancer cell-induced inflammation, as reflected by the sizes of spleens. That is, WWOX inhibits splenomegaly, whereas p53 fails to do so [76]. WWOX is most effective in suppressing cancer growth, and p53 less effective [76].

When in combination, p53 strongly abolishes the biological effects of WWOX, including cancer suppression and inhibition of inflammation (or causing splenomegaly). Most strikingly, cancer-regulated protein aggregation in the brain is blocked by WWOX (Figure 3). Again, p53 antagonizes WWOX in increasing protein aggregation. The p53/WWOX-cancer mice exhibit AD pathologies, including BACE (Beta-Secretase 1) upregulation, APP degradation, tau tangle formation, and amyloid β generation in the brain and lung [74]. The likely mechanism for the functional antagonism is that p53/TIAF1 blocks WWOX-mediated inhibition of inflammatory response and protein aggregation [76].

## 10. Concluding Remarks

Despite more than 100 years of research in AD, there are still no effective therapeutic drugs available to cure the disease [1,2,3,7]. Nowadays, more than 36.5 million persons worldwide are living with AD, and this may increase to 65.7 million in 2030 and to 115.4 million in 2050 [1,2,3,7]. Therefore, AD is declared as “global public health priority” by the World Health Organization (WHO) and is one of the greatest medical care challenges in the world [1,2,3,7].

It is generally considered that there is an inverse relationship between the development of cancer and neurodegeneration in vivo [92,93]. It is proposed that PIN1 and p53 play key roles to the inverse relationship. Nonetheless, biochemical basis provided by gene chip analyses revealed that many signaling pathways are shared by both cancer and neurodegeneration, including DNA damage, cell cycle aberrations, inflammation, immunity, and oxidative stress. Furthermore, specific genes are mutated or altered during the progression of cancer and AD such as α-synuclein, PTEN and PINK1 [92,93]. Despite the observations, it is difficult to predict and decipher the key proteins that run in a parallel or an opposite manner to manipulate the outcome of cancer and/or neurodegeneration.

One of the proteins that runs in parallel in promoting cancer and neurodegeneration is pS14-WWOX [28,81]. pS14-WWOX strongly enhances the progression of cancer and neurodegeneration. Inhibition of Ser14 phosphorylation by Zfra1-31 or Zfra4-10 peptide abolishes cancer growth and AD progression [28,81].

### 10.1. pS14-WWOX Is Linked to AD Progression 

WWOX exhibits a plethora of functional properties in vivo. WWOX restricts cancer growth and AD progression, induces immune cell differentiation [35,53,54], and blocks bacterial and viral infections [94]. pY33-WWOX exerts apoptosis and tumor suppression when overexpressed in vivo and in vitro. pY33-WWOX also integrates the signaling of Hyal-2/WWOX/Smad4 for TGF-β1 and hyaluronan, Wnt, and many other signal pathways [7,32,33,34,35,36,37,49,50]. WWOX phosphorylation at Tyr33 is reduced under certain conditions. In the instance of abnormal development of hematopoietic diseases and malignancies, calcium ionophore, and phorbol ester can force the differentiation of T lymphoblastic leukemia cells. The event involves a newly identified IκBα/WWOX/ERK signaling to drive leukemia cell differentiation, in which WWOX phosphorylation at Ser14 is required [35,53]. Normal T cell differentiation also needs pS14-WWOX.

Unfortunately, pS14-WWOX is significantly upregulated in the lesions of cancer [81] and AD hippocampus and cortex [28]. That is, as the disease is in progression, pY33-WWOX disappears and pS14-WWOX is upregulated to promote tumor growth and AD progression [28,81]. We believe that pS14-WWOX is likely to control the progression of other diseases.

### 10.2. Zfra and Zfra-Activated Z Cells for AD Therapy

Zfra4-10 and Zfra1-31 peptides are of therapeutic potential in blocking AD progression [28]. Two potential mechanisms are involved. First, Zfra dramatically suppresses S14 phosphorylation in WWOX (>90%), which leads to blockade of AD progression in 3xTg and *Wwox* heterozygous mice [28]. The full-length Zfra is only 31 amino acid [43,95]. As short as 7 amino acids, Zfra4-10 (RRSSSCK) is potent in cancer suppression and restoring memory loss [28,81]. Similar results were also observed using the full-length Zfra peptide. 

Second, exogenous full-length Zfra1-31 and Zfra4-10 peptides induce memory anticancer response via expansion of Zfra-reactive spleen Z lymphocytes [28,57,81]. We have first identified Z cells and showed that activated Z cells confer cancer resistance in recipient mice [28,57,81]. Zfra binds the membrane Hyal-2, and then recruits WWOX and Smad4 for gene transcription [28,57,81]. Without prior encountering with cancer antigens, Zfra-activated Z cells are able to recognize many cancer cell types and suppress their growth. Clonal expansion of activated Z cells and their killing of cancer cells can be seen in vitro [57]. Naïve Z cells cannot attack and kill cancer cells. The chances of Zfra-activated Z cells in blocking AD-related seizure and memory loss are strong.

### 10.3. Manipulating the Binding Strength between WWOX and Binding Partner Proteins to Limit Disease Progression

Supporting evidence showed that the stronger the binding strength between WWOX and its partner proteins, the better the extent of cancer suppression [57]. By the same token, WWOX is expected to limit AD progression effectively if its binding with partner proteins is strong. For example, WWOX needs to effectively bind tau and TPC6A∆ and control the function of tau-hyperphosphorylating enzymes [41,69,70]. Unfortunately, the level of WWOX starts to decline from middle age. Another conundrum is that endogenous WWOX is functionally inactivated by cytosolic Zfra [43]. That is, instead of using WWOX peptides for therapy, Zfra peptides are reliable and nontoxic and exhibit its activity in blocking AD progression and the growth of tumors [28,57,79]. While Zfra activates Z cells to block cancer growth, utilization of activated Z cells in treating seizure, WOREE, and AD development is expected to be promising.

## 11. Conclusions and Perspectives for the Future

In conclusion, WWOX and its binding proteins play a critical role in limiting inflammation and associated AD progression. The stronger the binding, the better the inhibition of inflammation and suppression of AD symptoms. Presumably, when there is an elevated intracellular Zfra, Zfra binds WWOX and the Zfra/WWOX complex is subjected to degradation. Consequently, free, excessive p53 and other WWOX-binding proteins start to exert inflammatory reactions [57,76].

Despite WWOX being a defined risk factor for AD [38], functional WWOX is needed to support the development of neural system and maintain normal neuronal physiology. Presence of dysfunctional WWOX, loss of WWOX, or excessive WWOX causes neurodegenerative diseases and neuronal death such as in AD [7,28,41], WOREE [60,61,62,63,69,70,71,72,73], sciatic nerve damage [42,96], retinal degeneration [68], and Parkinson’s disease [97].

Ectopic p53 and WWOX mediate apoptosis in a synergistic manner in vitro [31,53,80], whereas functional antagonism between these two proteins may occur in vivo. This leads to inflammatory splenomegaly and brain protein aggregation for AD in vivo. That is, an inflammatory reaction may occur as a result of an intracellular battle between p53 and WWOX. In principle, WWOX is a stronger inhibitor for inflammation and AD progression than p53. Under aberrant TGF-β/Smad/TIAF1 signaling [27], TIAF1 undergoes polymerization and causes cytoplasmic localization of Smads, WWOX, and p53. These proteins then become functionally inactivated. In the presence of excessive TGF-β1 in the microenvironment, aberrant TGF-β/Smad signaling occurs. Furthermore, p53 and TIAF1 together block WWOX or Smad4-regulated SMAD promoter activation. We do not exclude the possibility that p53/TIAF1/WWOX triad becomes aggregated in the brain and contributes to aggregation of tau and amyloid beta of the AD pathologies.

Despite several decades of global efforts, there are still no effective drugs available in curing AD. A recent monoclonal antibody-based drug Aducanumab for AD treatment has been approved by FDA [98]. Nonetheless, this has sparked numerous controversies. In the Table 1, we propose a few outstanding questions, along with suggested future research directions. We believe that these advanced concepts are good for developing strategies in drug design for curing AD.

## Figures and Tables

**Table 1:** Outstanding questions and future research directions.

Outstanding Questions	Suggested Future Research Directions
1. WWOX is potent in blocking inflammation and preventing protein aggregation [28,74,79]. Is there a feasible approach to use autologous normal cells for stably expressing WWOX so as to suppress inflammation and AD progression?	Cell therapy for reinstalling functional WWOX would provide a strong anti-inflammatory microenvironment in vivo. Autologous cells can be engineered to stably express functional WWOX and reinstalled in a non-brain area to shut down chronic inflammation in the brain.
2. Zfra effectively blocks cancer growth and inhibits AD progression by blocking chronic systemic inflammation [74]. However, Zfra inactivates WWOX by degradation via an unknown proteolytic mechanism. Can WWOX be replaced by exogenous Zfra peptide in mitigating AD progression?	Therapeutic peptides composed of both short Zfra and WWOX amino acid sequences can be made and tested for their efficacy in blocking AD progression and cancer growth in vivo. These peptides are expected to block seizure associated with WWOX deficiency-related syndromes such as WOREE in newborns. The peptides are not expected to be immunogenic to cause inflammatory response in vivo.
3. Chronic inflammation induces protein aggregation in the lung. Do lung protein aggregates accelerate the formation of protein polymerization and plaque formation in the brain?	Synthetic Zfra4-10 peptide can be used to eradicate lung protein aggregation in 3xTg mice. Concurrent restoration of memory loss in these mice is expected to be achieved also.
4. Activated Z cells suppress and prevent cancer growth [28]. Can autologous Z cell therapy be effective in preventing and blocking AD progression?	While Zfra activates Z cells in vivo, autologous activated Z cells can be purified and used for cell therapy. This will benefit patients suffering seizure, AD and other neurodegenerative diseases.

## Data Availability

Not available for this review article, except data for Figure 2 are at https://hbatlas.org/pages/hbtd; last visit on 25 June 2021.
